# Tenosynovial Giant Cell Tumor of the Cervical Spine: Case Report and Review of the Literature

**DOI:** 10.7759/cureus.12232

**Published:** 2020-12-23

**Authors:** Meena Thatikunta, Mohammed Nuru, Ashley E Mathew, Thomas J Altstadt

**Affiliations:** 1 Neurosurgery, University of Louisville Hospital, Louisville, USA; 2 Neurosurgery, University of Louisville School of Medicine, Louisville, USA; 3 Pathology, University of Louisville Hospital, Louisville, USA

**Keywords:** tenosynovial, giant cell tumor, cervical spine, trauma, spine oncology, diffuse type

## Abstract

Tenosynovial giant cell tumor (TGCT) is a rare entity that is not well described in the neurosurgical literature. We present a case of a 37-year-old woman with a diffuse subtype TGCT of the cervical spine, affecting the left cervical 6-7 facet joint, with co-incidental cervical trauma. Initial management consisted of subtotal resection and cervical stabilization with cervical 6 to 7 laminectomy, and cervical 4 to thoracic 2 posterior instrumented fusion. Gross total resection was achieved at a later date with a plan for postoperative radiation to prevent a recurrence. The patient was lost to follow-up for radiation treatment and returned 2.5 years later with minor symptoms and recurrence at the surgical site.

## Introduction

Tenosynovial giant cell tumors (TGCTs) are rare tumors originating from the tendon sheath of joints, bursae, or joint synovia. Generally, TGCTs are found in the phalangeal joints, hips, and knees and thus are well known in the orthopedic literature [1,2]. These tumors are rarely found in the spine [3,4]. Here we present a case report of the neurosurgical management of TGCT diffuse subtype in the cervical spine and a review of the literature about cases of spinal TGCT.

## Case presentation

A 37-year-old woman presented to the emergency department after being ejected from an all-terrain vehicle while unhelmeted. At the time of presentation, she was hemodynamically unstable due to extra-corporeal blood loss from scalp degloving. Other injuries included a left temporal bone fracture, skull base fractures, and a thoracic 6 compression fracture. She was asymptomatic from a neurosurgical standpoint and had no known history of malignancy. Her neurological examination was unremarkable.

**Figure 1:**
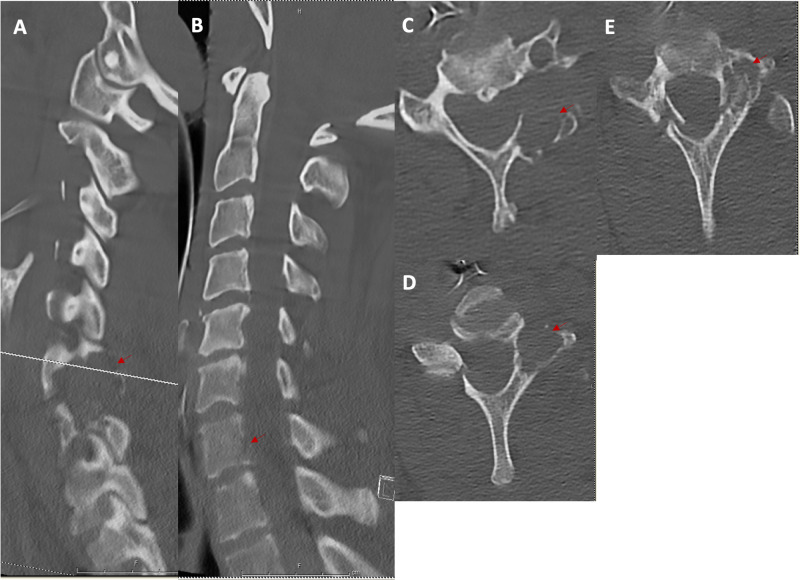
Cervical CT of traumatic fracture and osteolytic lesion (A) Left parasagittal cervical CT demonstrating osteolytic lesion (red arrows) at cervical 6 and 7 levels. (B) Mid-sagittal cervical CT cervical 7 vertebral body fracture without compression of the spinal cord. (C) Axial CT at the level of cervical 6 demonstrating osteolytic lesion originating at the left facet joint and abutting the left vertebral foramen. (D) Axial CT at the level of cervical 7 demonstrating an osteolytic lesion originating at the left facet joint with visualization of right laminar fracture and partial visualization of left laminar fracture. (E) Axial CT at the level of cervical 7 better demonstrating left laminar fracture CT: computed tomography

**Figure 2:**
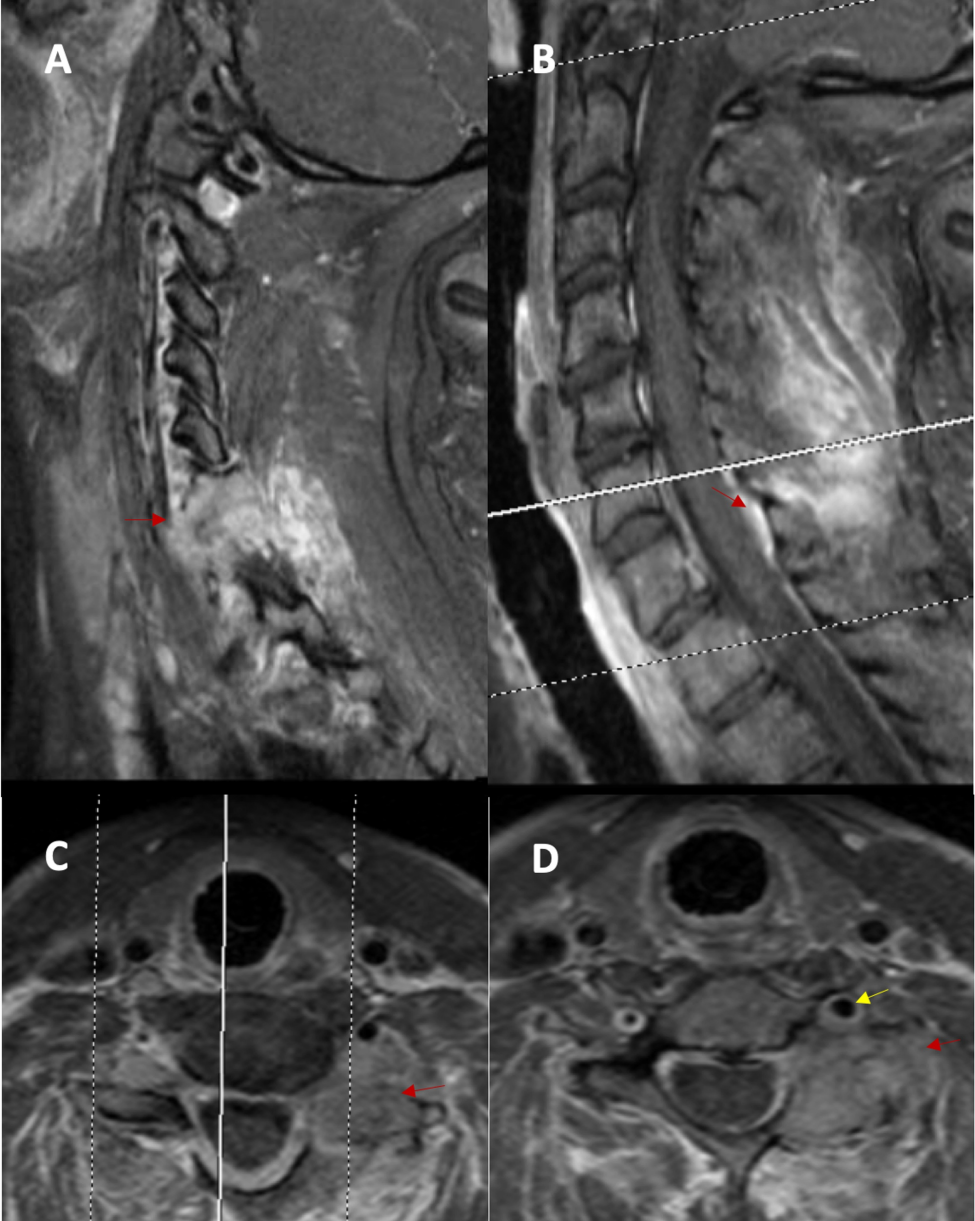
MRI cervical with and without contrast further characterizing mass (A) Left parasagittal cervical MRI with contrast demonstrating heterogeneously enhancing mass (red arrows) originating from the facets with extension into the soft tissues of the neck. (B) Sagittal MRI with contrast demonstrating extradural mass of the posterior elements with extension into the soft tissues. (C) Axial MRI with contrast at the level of C6 shows mass involvement with the left facet and abuttal of the left vertebral artery. (D) Axial MRI with contrast at the level of C7 showing mass involvement of the left facet and abuttal with possible encasement of the left vertebral artery (yellow arrow) MRI: magnetic resonance imaging

The initial CT spine scan demonstrated a cervical 7 vertebral body fracture and bilateral laminar fractures. An incidental C6/7 left facet osteolytic mass was also found; the mass abutted the transverse foramen without evidence of invasion (Figure 1). MRI revealed a heterogenous contrast-enhancing extradural mass originating in the left cervical 6 and 7 facet joint with extension into the soft tissues of the neck and epidural space without cord compression. The mass encircled the left vertebral artery (Figure 2). CT angiography (CTA) of the neck showed a dominant left vertebral artery. A metastatic workup was negative.

Initial management

The patient first underwent surgical repair for scalp avulsion and degloving without complication. On our assessment, we deemed the cervical spine unstable due to the two-level facet destruction from the mass. We planned for surgical stabilization, fusion, and debulking of the tumor with the extent of resection to be based on intraoperative pathology.

Intervention

**Figure 3:**
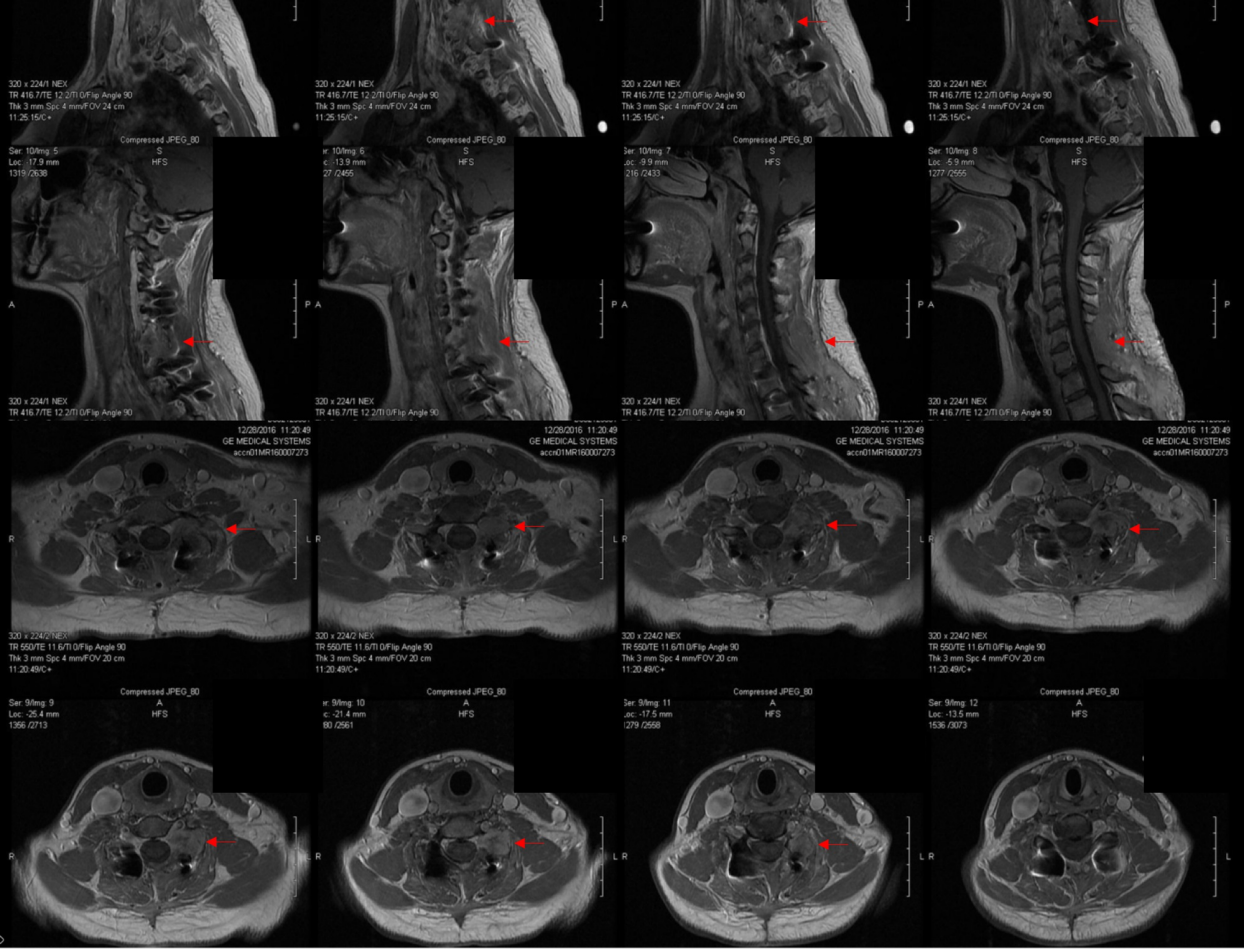
Postoperative MRI after the initial surgery Postoperative contrasted sagittal and axial MRI after the initial surgery shows a subtotal resection with residual tumor (red arrows) at the facet and near the left vertebral artery MRI: magnetic resonance imaging

A cervical 6 to 7 laminectomy, with cervical 4 to thoracic 2 posterior instrumented fusion, and tumor debulking was performed. Tumor biopsy samples were sent to pathology for assessment. Intraoperative pathology was inconclusive with concern for possible plasmacytoma or melanoma. We resected the majority of the tumor, while a small portion that was deep and adjacent to the vertebra was left behind. This portion of the tumor was separated away from the neural elements. Biopsy samples were sent out from our institution for further pathological assessment. Figure 3 shows the postoperative MRI. The patient was discharged home on postoperative day four with cervical and lumbar braces, with a plan to follow up in the clinic for assessment. The pathological report revealed TGCT (see Pathology section below).

Follow-up 

Anterior/posterior and lateral cervicothoracic X-ray at the one- and two-month follow-ups showed good hardware position from C4 to T2. The patient had a normal physical exam and was neurologically intact. Updated cervical MRI at the three-month follow-up showed residual tumor growth and plans were made to discuss treatment at the interdepartmental tumor board.

Pathology

**Figure 4:**
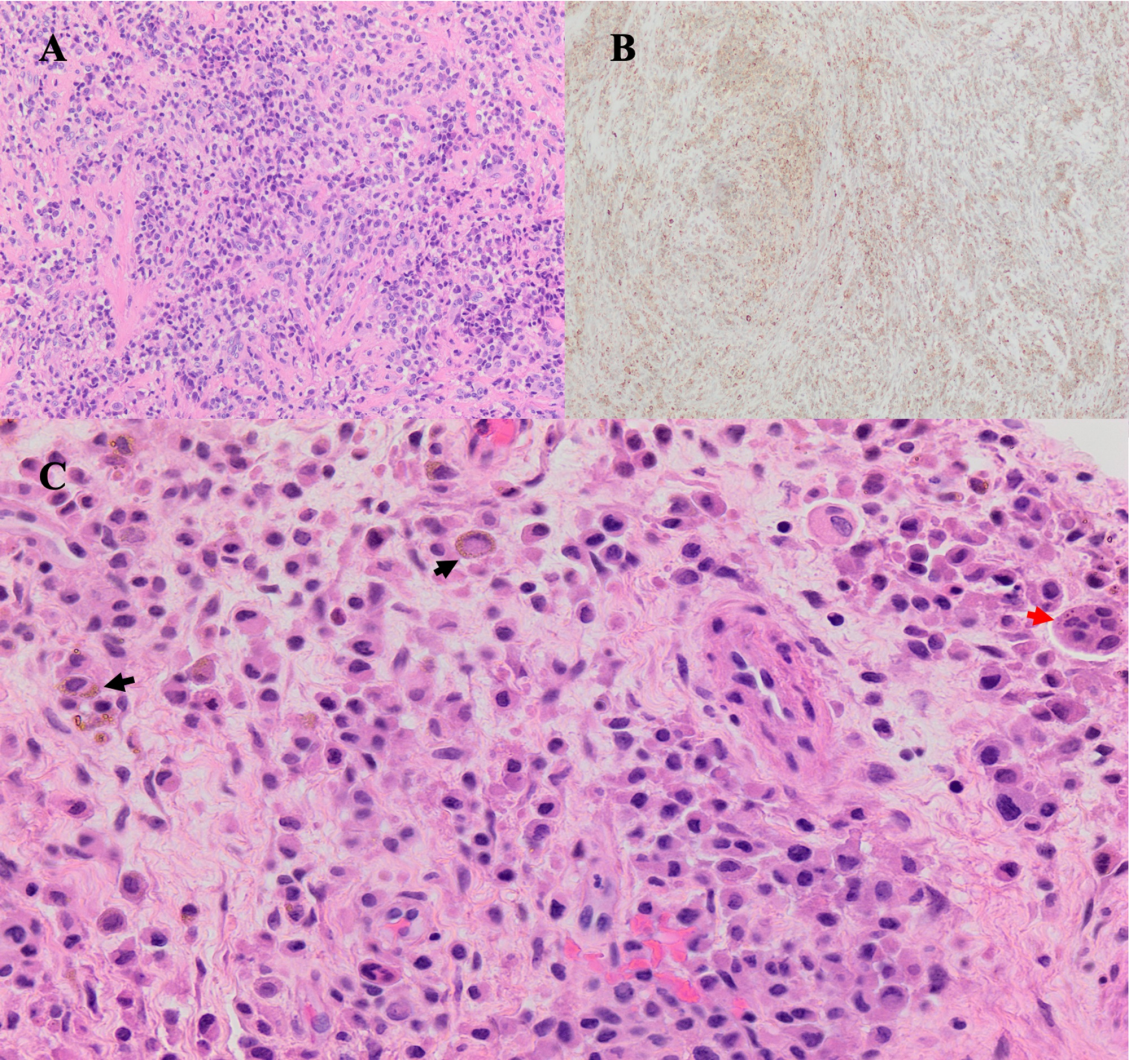
Pathologic staining of the tumor (A) H&E staining shows dense lymphohistiocytic proliferation with no atypia or mitotic figures identified. (B) CD68 immunostaining reveals diffuse positivity reflecting a mononuclear infiltrate characteristic of giant cell tumors. (C) H&E staining shows multinucleated giant cells (red arrow) along with pigment-laden macrophages (black arrow) H&E: hematoxylin and eosin

Immunostaining of intraoperative tumor samples revealed lymphohistiocytic infiltration, multinucleated giant cells with pigment-laden macrophages, mononuclear infiltrate, and positive CD64 and CD45 staining (Figure 4). Several weeks later, the final pathology confirmed the diagnosis of TGCT, diffuse type.

Second operation

The patient’s case was presented at our inter-departmental tumor board. Due to the locally aggressive nature of diffuse-type giant cell tumors overall, we elected to conduct a second operation with the goal of total resection of the tumor to be followed by postoperative radiation. Preoperative planning included a balloon test occlusion of the left vertebral artery in the event the vertebral artery needed to be sacrificed or was injured during the operation. The patient failed the balloon test occlusion and the dominant status of the left vertebral artery was confirmed.

**Figure 5:**
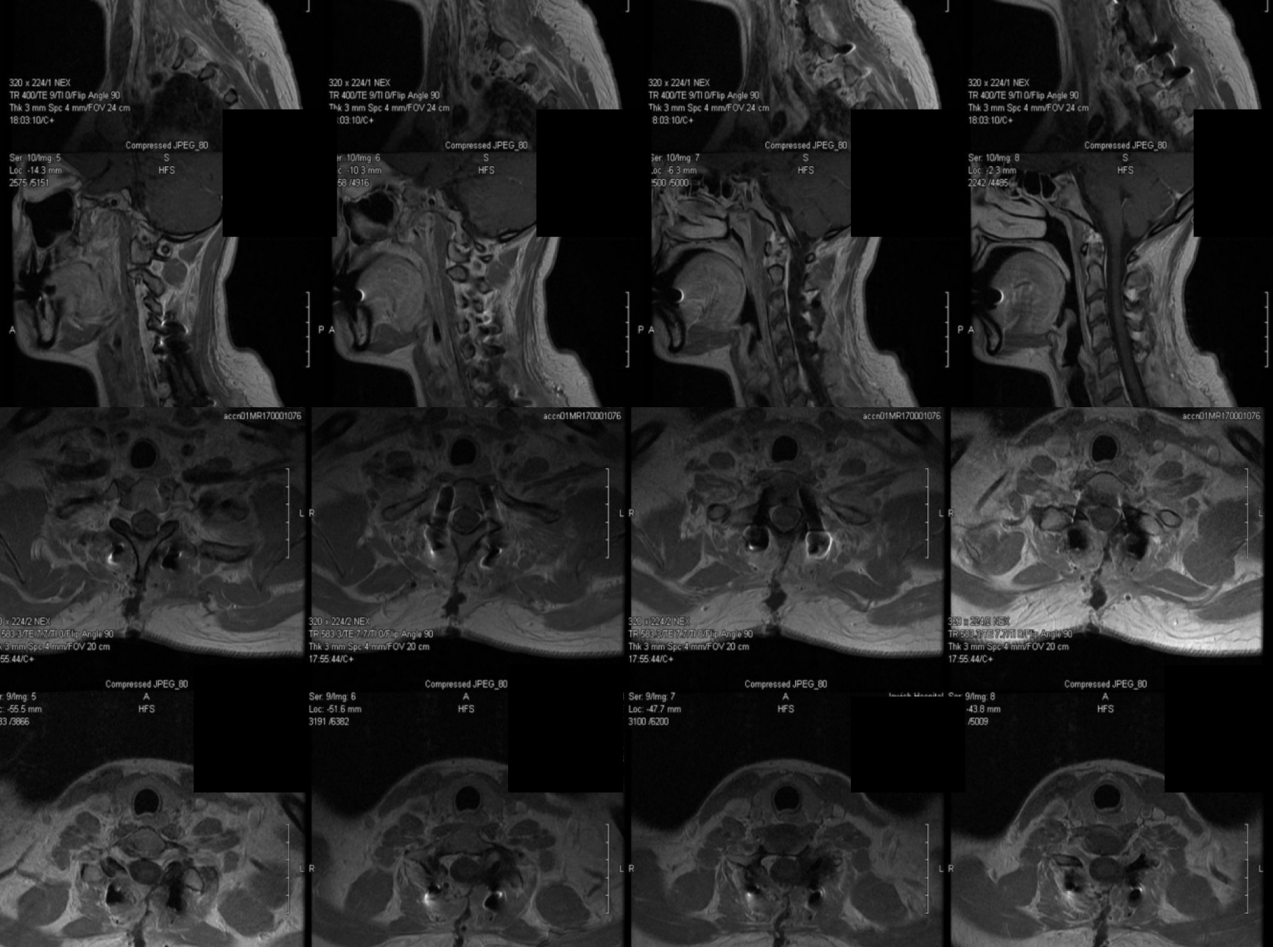
Postoperative MRI after the second surgery Postoperative contrasted MRI sagittal and axial after the second operation demonstrating gross total resection of the tumor MRI: magnetic resonance imaging

After prepping the posterior cervical region, the previous incision was open with a scalpel down to the fascia. The paraspinous muscle was dissected off the spinous process, lamina, and facet joints on the left side. The previous C4 to T2 rod was identified and the locking caps and rod removed. We then removed the screws at C5 and T1, allowing us to easily identify the recurrent tumor. The tumor was found to be epidural in nature and exited along the cervical 7 and 8 nerve roots. The tumor was carefully dissected from the lateral thecal sac, exiting nerve roots at C7 and C8, and the vertebral artery. Gross total resection was achieved. Figure 5 shows a postoperative MRI.

Despite the initial plan for postoperative radiation, the patient was lost to follow-up for an extended period of time. The patient returned to the clinic after 2.5 years with minor residual numbness in the left 5th digit. MRI at this time showed recurrence at the operative site. CT scan was performed and showed no evidence for bony destruction. Our interdepartmental tumor board reviewed the case and recommended serial imaging.

## Discussion

While well described for non-spine cases in the orthopedic literature, tenosynovial giant cell is rarely found in the spine and is not well described in the neurosurgical literature [3,4]. Table 1 reviews all the reported cases (English-language) of tenosynovial giant cell in the spine, with long-term follow-ups where available. Our patient was of the characteristic age (37 years) at presentation and her tumor was found incidentally through a trauma workup. Giant cell tumors affect patients in the third and fourth decades of life. TGCT is often asymptomatic or painful with a history of minor antecedent trauma; some contend that trauma is related to its pathogenesis [5]. In this patient’s case, management was complicated by the coexistence of traumatic cervical fractures, and she underwent two operations for definitive management: the first for stabilization and pathologic diagnosis and the second to accomplish gross total resection. It is worth mentioning however that 9-30% of giant cell tumor patients present with pathological fractures. Our patient's cervical fractures (inferior posterior aspect of C7 vertebral body) seemed largely traumatic in nature; however, cervical MRI showed destruction of transverse processes of C6 and C7 vertebral processes; so it is possible that some subclinical osteolytic weakening might have been a contributing factor. The incidence of pathological fractures in giant cell tumor patients (9-30%) is similar to the incidence of pathological fractures seen in metastatic spine patients (9-29%).

**Table 1:** Reported cases of tenosynovial giant cell tumors GTR: gross total resection; R: surgery performed but not indicated whether gross or subtotal resection achieved

Reference	Demographic	Chief complaint	Location	Resection	Postoperative sx	Recurrence
Cervical						
Blankenbaker et al. [6]	43, male	Trauma	C1 posterior arch	R		Unknown follow-up
Bui-Mansfield et al. [7]	25, male	Trauma	C4			Unknown follow-up
Dingle et al. [8]	27, female	Neck pain	C6/C7 facet	R		None (2 yrs)
Okutan et al. [9]	65, male	Neck pain and lower extremity weakness	C7	GTR		None (6 mos)
Yamada et al. [10]	63, female	Incidental mass	C1	GTR		None (3 yrs)
Mahmood et al. [11]	42, female	Trauma	C6/C7	R		Unknown follow-up
Thoracic						
del Carmen Baena-Ocampo et al. [12]	17, male	Back pain/myelopathy	T9 pedicle			Unknown follow-up
Doita et al. [13]	26, male	Back pain	T7-T8 facet	R		None (2 yrs)
Lumbar						
Campbell et al. [14]	54, female	Back pain/radiculopathy	L4/L5	R	None (3 yrs)	
Hsieh et al. [15]	39, male	Back pain	L2-L3	GTR		Unknown follow-up
Weidner et al. [16]	48, female	Back pain	L5/L6	R		4 mos at L4-L5 - GTR; symptom-free (2 mos)

Generally, TGCTs are benign, and rarely exhibit malignant behavior [1,2]. The recurrence rate in the spine is estimated at 17-48% [16,17]. The diffuse type is prone to pathologic misdiagnosis [18]. The diffuse type is an aggressive subtype causing greater bony destruction and local growth. The recurrence rate for diffuse subtypes is higher but the rate differs among reports, ranging from 17 to 71% [16,19].

Risk factors for recurrence of TGCT are not well codified due to the rare nature of the tumor. Risk factors may include: (1) diffuse subtype, (2) bone erosion, (3) neurovascular involvement, and (4) size of >2 centimeters [20]; this patient was noted to have all four risk factors for recurrence.

Maximal resection in diffuse subtypes offers the best chance for reducing recurrence (7.7% recurrence rate) [20]. Some have emphasized the need to remove the entire synovium to prevent recurrence [5]. Giant cell tumors are radiosensitive, and radiation is generally recommended for subtotal resection; however, its role is controversial [9,10]. Due to the number of risk factors present for recurrence in this case, despite the gross total resection, postoperative radiation was planned should recurrence occur. This patient was lost to follow-up and did not receive postoperative radiation. The two-year follow-up MRI showed recurrence.

## Conclusions

TGCT is a benign tumor of the tendon sheath, bursae, and synovium that is rarely found in the spine. This tumor is rarely described in the neurosurgical literature. In this report, we presented a complex case of cervical stabilization and gross total resection of a locally aggressive diffuse-type giant cell tumor with abuttal of a dominant left vertebral artery.

## References

[1] Jaffe HL (1941). Pigmented villonodular synovitis, bursitis and tenosynovitis. Arch Pathol.

[2] Llauger J, Palmer J, Rosón N, Cremades R, Bagué S (1999). Pigmented villonodular synovitis and giant cell tumors of the tendon sheath: radiologic and pathologic features. AJR Am J Roentgenol.

[3] Giannini C, Scheithauer BW, Wenger DE, Unni KK (1996). Pigmented villonodular synovitis of the spine: a clinical, radiological, and morphological study of 12 cases. J Neurosurg.

[4] Lavrador JP, Oliveira E, Gil N, Francisco AF, Livraghi S (2015). C1-C2 pigmented villonodular synovitis and clear cell carcinoma: unexpected presentation of a rare disease and a review of the literature. Eur Spine J.

[5] Oe K, Sasai K, Yoshida Y, Ohnari H, Iida H, Sakaida N, Uemura Y (2007). Pigmented villonodular synovitis originating from the lumbar facet joint: a case report. Eur Spine J.

[6] Blankenbaker DG, Tuite MJ, Koplin SA, Salamat MS, Hafez R (2008). Tenosynovial giant cell tumor of the posterior arch of C1. Skeletal Radiol.

[7] Bui-Mansfield LT, Youngberg RA, Coughlin W, Chooljian D (1996). MRI of giant cell tumor of the tendon sheath in the cervical spine. J Comput Assist Tomogr.

[8] Dingle SR, Flynn JC, Flynn JC Jr, Stewart G (2002). Giant-cell tumor of the tendon sheath involving the cervical spine. A case report. J Bone Joint Surg Am.

[9] Okutan O, Solaroglu I, Ozen O, Saygili B, Beskonakli E (2012). Tenosynovial giant cell tumor in the cervico-thoracic junction. Turk Neurosurg.

[10] Yamada S, Oshima K, Hamada K (2016). Giant cell tumor of the tendon sheath arising from a membrane surrounding the posterior arch of C1: a case report. Spine J.

[11] Mahmood A, Caccamo DV, Morgan JK (1992). Tenosynovial giant-cell tumor of the cervical spine. Case report. J Neurosurg.

[12] del Carmen Baena-Ocampo L, Rosales Olivares LM, Arriaga NM, Izaguirre A, Pineda C (2009). Pigmented villonodular synovitis of thoracic facet joint presenting as rapidly progressive paraplegia. J Clin Rheumatol.

[13] Doita M, Miyamoto H, Nishida K, Nabeshima Y, Yoshiya S, Kurosaka M (2005). Giant-cell tumor of the tendon sheath involving the thoracic spine. J Spinal Disord Tech.

[14] Campbell AJ, Wells IP (1982). Pigmented villonodular synovitis of a lumbar vertebral facet joint. J Bone Joint Surg Am.

[15] Hsieh YC, Chen WY, Hsieh TY, Chan WP (2012). Pigmented villonodular synovitis of the lumbar spine. J Clin Rheumatol.

[16] Weidner N, Challa VR, Bonsib SM, Davis CH Jr, Carrol TJ Jr (1986). Giant cell tumors of synovium (pigmented villonodular synovitis) involving the vertebral column. Cancer.

[17] Yener U, Konya D, Bozkurt S, Ozgen S (2010). Pigmented villonodular synovitis of the spine: report of a lumbar case. Turk Neurosurg.

[18] Goldblum J, Weiss S, Folpe AL (2013). Enzinger and Weiss's Soft Tissue Tumors. https://www.elsevier.com/books/enzinger-and-weisss-soft-tissue-tumors/goldblum/978-0-323-08834-3.

[19] Bruecks AK, Macaulay RJ, Tong KA, Goplen G (2001). November 2000: 13 year old girl with back pain and leg weakness. Brain Pathol.

[20] Gouin F, Noailles T (2017). Localized and diffuse forms of tenosynovial giant cell tumor (formerly giant cell tumor of the tendon sheath and pigmented villonodular synovitis). Orthop Traumatol Surg Res.

